# 

**Published:** 1912-12

**Authors:** 


### The Library of the Bristol Medico-Chirurgical Society.

*The following donations have been received since the publication of the List in September:*

*November 30th*, 1912.

| | |
|---|---|
| E. W. Hey Groves (1) .. .. .. .. | 1 volume. |
| The Middlesex Hospital (2) .. .. .. | 1 ,, |
| P. A. Opie, M.B. (3) .. .. .. .. | 1 ,, |
| Arthur B. Prowse, M.D. (4) .. .. .. | 8 volumes. |

Framed pictures have been presented by the late Dr. Robert Fletcher and Mr. L. M. Griffiths.

### EIGHTY-SIXTH LIST OF BOOKS.

The titles of books mentioned in previous lists are not repeated.

The figures in brackets refer to the figures after the names of the donors, and show by whom the volumes were presented. The books to which no such figures are attached have either been bought from the Library Fund, or received through the *Journal*.

**Adami and J. McCrea, J. G.** *A Text-Book of Pathology* .. .. .. 1912

*Anatomy* (Catechism Series), Part I .. .. .. .. .. .. .. .. [N.D.]

**Armstrong and J. M. Fortescue-Brickdale, H. G.** *Manual of Infectious Diseases occurring in Schools* .. .. .. 1912

**Bayly, H. W.** .. *Clinical Pathology of Syphilis* .. .. .. 1912

**Bedford, C. H.** .. *Clinical Handbook of Urine Analysis* (4) 2nd Ed. 1904

**Bennett, R. R.** .. *Materia Medica and Pharmacy* .. 2nd Ed. 1912

**Burghard, Sir W. W. Cheyne and F. F.** *A Manual of Surgical Treatment.* New Edition. (Revised by T. P. Legg and A. Edmunds) .. Vol. III 1912

**Bury, J. S.** .. .. *Clinical Medicine* (Edited by J. S. Bury and A. Ramsbottom) .. .. .. .. 3rd Ed. 1912

**Carter, A. H.** .. *Elements of Practical Medicine* .. 10th Ed. 1912

**Cheadle, W. B.** .. *Rheumatic State in Childhood* .. .. (4) 1889

**Cheyne and F. F. Burghard, Sir W. W.** *A Manual of Surgical Treatment.* New Edition. (Revised by T. P. Legg and A. Edmunds) .. Vol. III 1912
**Choyce, C. C.** [Ed.] *A System of Surgery* .. .. .. Vol. II 1912
**Collie, A.** .. .. *Small-pox and its Diffusion* .. .. .. .. .. 1912
**Craig, W.** .. .. *Manual of Materia Medica* .. (4) 5th Ed. 1887
**Creighton, C.** .. *Bovine Tuberculosis in Man* .. .. .. .. (4) 1881
**Emery, W. D'E.** .. *Clinical Bacteriology and Hæmatology* 4th Ed. 1912
**Fearis, W. H.** .. *Treatment of Tuberculosis by Immune Substances* .. .. .. .. .. .. .. .. .. 1912
**Fortescue-Brickdale, H. G. Armstrong and J. M.** *Manual of Infectious Diseases occurring in Schools* .. 1912
**Frazer, Elizabeth T.** *A Manual of Immunity* .. .. .. .. .. .. 1912
**Gabbett, P. C.** .. *Manual for Women's Voluntary Aid Detachments* .. .. .. .. .. .. .. .. .. .. [N.D.]
**Geddes, G.** .. .. *Statistics of Puerperal Fever* .. .. .. .. .. 1912
**Gibbon, I. G.** .. *Medical Benefit in Germany and Denmark* .. 1912
**Gibbs, J. H.** .. *Extraction of Teeth* .. .. .. .. .. .. .. .. 1912
**Gibson, G. A.** .. *Life of Sir William Tennant Gairdner* .. .. 1912
**Gros, A.-P.** .. .. *Traitement de Maladies de l'Estomac* .. (3) 1898
**Hawthorne, C. O.** *Studies in Clinical Medicine* .. .. .. .. .. 1912
**Hawthorne, C. O.** *Forensic Medicine and Toxicology* 3rd Ed. 1912
**Hewitt, Sir F. W.** *Anæsthetics and their Administration* 4th Ed. 1912
**Hollander, B.** .. *The First Signs of Insanity* .. .. .. .. .. [1912]
**Hutt, C. W.** .. .. *Hygiene for Health Visitors* .. .. .. .. .. 1912
**Kirkby, W.** .. .. *Practical Prescribing and Dispensing* .. (4) 1904
**Knopf, S. A.** .. *Prophylaxis, and Treatment of Pulmonary Tuberculosis* .. .. .. .. .. .. .. .. (4) 1899
**McCrae, J. G. Adami and J.** *A Text-Book of Pathology* .. .. 1912
**McKendrick, W. Robertson and A.** *Public Health Law* .. .. 1912
**McKenzie, D.** .. *Diseases of the Nose, Throat and Ear* (4) 1908
**Martindale and W. W. Westcott, W. H.** *The Extra Pharmacopœia* 2 Vols. 15th Ed. 1912
**Miles, C. H.** .. .. *An Historical Outline of Ambulance* .. .. [1912]
**Mitchell, S. W.** .. *Some Recently discovered Letters of William Harvey* .. .. .. .. .. .. .. .. .. .. .. 1912
*Operative Surgery* (Catechism Series) Pt. 1 .. .. .. .. .. .. [N.D.]
**Porter, W. G.** .. *Diseases of the Nose, Throat and Ear* .. .. 1912
**Pye, W.** .. .. *Surgical Handicraft* (Edited by W. H. Clayton-Greene) .. .. .. .. .. 6th Ed. 1912
**Rawling, L. B.** .. *The Surgery of the Skull and Brain* .. .. 1912
**Rice, A. G.** .. .. *Medical Inspection of Schools* .. .. .. .. 1912
**Robertson and A. McKendrick, W.** *Public Health Law* .. .. .. 1912
**Rolleston, H. D.** .. *Diseases of the Liver, Gall-Bladder and Bile Ducts* .. .. .. .. .. .. .. .. .. .. 1912
**Sawyer, Sir J.** .. *Insomnia: its Causes and Treatment* 2nd Ed. 1912
**Schlaginweit, F.** .. *Nierentuberkulose* .. .. .. .. .. .. .. .. 1912
**Schottelius, M.** .. *Bacteria* (Tr. by H. Geoghegan) .. 2nd Ed. 1912

*Secret Remedies, More* .. .. .. .. .. .. .. .. .. .. .. .. 1912
**Sequeira, J. H.** .. *The Treatment of Ringworm* .. .. .. .. .. 1912
**Stoddart, W. H. B.** *Mind and its Disorders* .. .. .. 2nd Ed. 1912
*Surgery* (Catechism Series) .. .. .. .. .. .. .. .. Pts. III, IV [N.D.]
**Vincent, R.** .. .. *The Nutrition of the Infant* .. .. 4th Ed. 1913
**Vincent, S.** .. .. *Internal Secretions and the Ductless Glands* .. 1912
**Westcott, W. H. Martindale and W. W.** *The Extra Pharmacopœia* 2 Vols. 15th Ed. 1912
**Whitby, C. J.** .. *The Doctor and his Work* .. .. .. .. .. 1912
**Woodwark, A. S.**.. *Manual of Medicine* .. .. .. .. .. .. 1912
**Yearsley, M.** .. .. *Causes Leading to Educational Deafness in Children* .. .. .. .. .. .. .. .. .. .. 1912
**Yeo, I. B.** .. .. *Health Resorts* .. .. .. .. .. .. .. (4) 1882

### TRANSACTIONS, REPORTS, JOURNALS, &c.

American Journal of Obstetrics, The .. .. .. .. Vol. LXV 1912

American Journal of the Medical Sciences, The .. Vol. CXLIII 1912

American Urological Society, Transactions of the .. Vol. VI 1912

Anuario Médico Sud-Americano .. .. .. .. .. .. .. .. 1912

Archives of the Middlesex Hospital .. .. .. (2) Vol. XXVII 1912

Archiv für klinische Chirurgie .. .. .. .. Vol. XCVII 1912

Boston Medical and Surgical Journal, The .. .. Vol. CLXVI 1912

British Medical Journal, The .. .. .. .. .. Vol. I for 1912

Clinical Journal, The .. .. .. .. .. .. Vol. XXXIX 1912

Congrès de l'Association internationale d'Urologie, Procès-Verbaux du 2me. .. .. .. .. .. .. .. .. .. .. .. .. .. .. 1912

Deutsche Zeitschrift für Chirurgie .. .. Vols. CXIII–CXVII 1912

Echo médical du Nord, L'. .. .. .. .. .. .. .. .. .. .. 1911

Edinburgh Medical Journal .. .. .. .. .. N.S., Vol. VIII 1912

Edinburgh Obstetrical Society, Transactions of the Vol. XXXVII 1912

Glasgow Medical Journal, The .. .. .. .. Vol. LXXVII 1912

Henry Phipps Institute, Sixth Annual Report of .. .. .. .. 1912

Hospitals—

  Philadelphia Hospital Reports .. .. .. .. .. Vol. VIII 1911

  St. Thomas's Hospital Reports .. .. .. .. Vol. XXXIX 1912

Jahreskurse für Arztliche Fortbildung .. .. .. .. .. .. (1) 1911

Journal of Experimental Medicine, The .. .. .. Vol. XV 1912

Journal of Medical Research, The .. .. .. .. Vol. XXVI 1912

Journal of Obstetrics and Gynæcology .. .. .. Vol. XXI 1912

Journal of the American Medical Association, The .. Vol. LVIII 1912

Lancet, The .. .. .. .. .. .. .. .. .. Vol. I for 1912

Library World, The .. .. .. .. .. .. .. Vol. XIV 1911–12

Medical Chronicle, The .. .. .. 4th Series, Vols. XXII, XXIII 1912

Medical Press and Circular, The .. .. Vols. CXLII, CXLIV 1911–12

Medical Record.. .. .. .. .. .. .. .. .. Vol. LXXXI 1912

Münchener medizinische Wochenschrift .. .. .. Bd. I für 1912

Nordiskt medicinískt Archiv .. .. .. .. .. .. .. .. 1910

| | | |
|---|---|---|
| Philippine Journal of Science | Vol. VI | 1911 |
| Post Graduate, The | Vols. XXV, XXVI | 1910–11 |
| Practitioner, The | Vol. LXXXVIII | 1912 |
| Progressive Medicine | Vol. II | 1912 |
| Revue de Chirurgie | Tom. XLV | 1912 |
| Revue hebdomadaire de Laryngologie | Tom. XXIII, Vol. I | 1912 |
| Royal Academy of Medicine in Ireland, Transactions of the | Vol. XXX | 1912 |
| Surgery, Gynecology and Obstetrics | Vol. XIV | 1912 |
| Zentrablatt für Chirurgie | Bd. I für | 1912 |
| Zentrablatt für Gynäkologie | Bd. I für | 1912 |
| Zentrablatt für innere Medizin | Bd. I für | 1912 |

### MEETINGS OF SOCIETIES.

### Bristol Medico-Chirurgical Society.

### Local Medical Notes.

---

### UNIVERSITY OF BRISTOL.

Students of the University have been successful in the following examinations :—

D.P.H. Bristol.—W. Pomeroy.

Conjoint Board of England.—*Chemistry :* J. H. Eglinton. *Physics :* E. Butler. *Medicine :* L. Page. *Surgery :* G. S. Applegate. *Midwifery :* C. Kingston, W. E. Neale.

L.D.S., R.C.S. Eng.—*Final Examination* (*Part I*): K. Batten, R. H. Basker. *Part II*: P. L. Ealand, J. W. Gilbert. *Mechanical Dentistry*: F. K. Hayman, E. W. Thomas, A. H. Virgin. *Dental Metallurgy*: A. H. Virgin, Marjorie J. White.

**Medical School Dinner.**—This function took place on November 21st, when Dr. Devis presided. Sir John Bland-Sutton was the Guest of the Evening.

An almost record number sat down to dinner, and later in the evening Dr. Devis, one of the Bristol representatives, gave an account of what took place at the recent Special Representative Meeting.

### APPOINTMENTS.

**Bristol Royal Infirmary.**—The following appointments have recently been made:—*Senior Resident Officer*: S. H. Kingston, M.B., Ch.B. *House Physician*: R. C. Clarke, M.B., Ch.B. *House Surgeon*: H. W. Goodden, M.B., Ch.B. *House Surgeon to Ear, Nose and Throat Department*: J. P. I. Harty, F.R.C.S., M.B., B.Ch.

**Bristol General Hospital.**—The following appointments have recently been made:—*Senior Resident Officer*: D. Wood, F.R.C.S. *House Surgeon*: R. F. Bolt, M.R.C.S., L.R.C.P. *House Physicians*: J. M. Lang, M.B., Ch.B. Glasgow, and D. W. Rintoul, M.B., Ch.B. St. Andrew's. *Casualty House Surgeon*: G. A. Smyth, B.A. Cantab., M.R.C.S., L.R.C.P.

**Royal Hospital for Sick Children and Women.**—The following appointments have recently been made:—*House Surgeons*: G. S. Griffiths, M.R.C.S., L.R.C.P., and W. A. Reynolds, M.B., Ch.B.

**Cossham Hospital.**—E. S. Abraham, M.R.C.S., L.R.C.P.

**Winsley Sanatorium.**—Charles J. Alexander, M.B., B.Ch., B.A.O., has been appointed Resident Medical Officer, *vice* Leonard Crossley, M.D., appointed Tuberculosis Officer, County of Wilts.

---

Captain W. T. McCowen.—"The Secretary of State for India has been pleased to request the Government of India to convey the thanks of His Majesty's Government to Captain W. T. McCowen, I.M.S., for the services he rendered on the occasion of the attack upon Acting-Consul Smart, near Kazerun, in December, 1911." Captain McCowen was at one time Junior House Surgeon and Ophthalmic House Surgeon at the Bristol Royal Infirmary.

### INDEX.

Abdominal pain, Causes of chronic recurrent, 282.
Alexander, Dr. D. A.—Raynaud's disease, 247.
Alimentary canal, Sensibility of the, 256.

Barber surgeons, The—Dr. G. Parker, 327.
Benzene derivatives, The relationship between toxicity and chemical reactivity in certain—Dr. O. C. M. Davis, 237.
Bismuth meals, 70, 253, 350.
**Books, Reviews of**—
Alderson, Dr. W. E.—Dental anæsthetics, 272.
Allbutt and Dr. H. D. Rolleston, Sir T. C. [Eds.]—A system of medicine, 74.
Allyl sulphide in treatment of tuberculosis and lupus—Dr. W. C. Minchin, 271.
Ambulance examination questions—Dr. D. M. Macdonald, 83.
Anæsthesia and analgesia—Dr. J. D. Mortimer, 160.
Anæsthetics, Dental—Dr. W. E. Alderson, 272.
Anæsthetics, Practical—H. E. G. Boyle, 173.
Anatomy, Practical—F. G. Parsons and Dr. W. Wright, 262.
Asthma, Bronchial—Dr. J. B. Berkhart, 267.
Bell, Dr. W. B.—The principles of gynæcology, 363.
Berkhart, Dr. J. B.—Bronchial asthma, 267.
Bidwell, L. A.—Minor surgery, 161.
Bone, The growth of—W. Macewen, 161.
Bones and joints, Deformities, including diseases of—A. H. Tubby, 264.
Bones and joints, Tuberculous diseases of—Sir W. W. Cheyne, 75.
Boyle, H. E. G.—Practical anæsthetics, 173.
Brockbank, Dr. E. M.—Children, their care and management, 366.
Bulletin of the committee for the study of special diseases, 171.
Burghard, Sir W. W. Cheyne and F. F.—Manual of surgical treatment, 162.
Candy, H.—Manual of physics for students of medicine, 83.
Carbonic-acid snow as a therapeutic agent—Dr. R. C. Low, 82.
Catalogue (Index-) of the library of the Surgeon-General's office, 273.
Cheyne, Sir W. W.—Tuberculous diseases of bones and joints, 75.

**Books, Reviews of** (*continued*)—
Cheyne and F. F. Burghard, Sir W. W.—A manual of surgical treatment, 162.
Children, their care and management—Dr. E. M. Brockbank, 366.
Chirurgisches vademecum — Dr. A. Schönwerth, 172.
Choyce, Dr. C. C. [Ed.]—A system of surgery, 74.
Clinical methods—Drs. R. Hutchison and H. Rainy, 270.
Consumption, Home-treatment of—Dr. H. W. Crowe, 268.
Crowe, Dr. H. W.—Consumption: treatment at home, 268.
Deaf child, The—Dr. J. K. Love, 266.
Deformities—A. H. Tubby, 264.
Dental caries, Prevention of—Dr. H. P. Pickerill, 273.
Dermatology, An introduction to—Dr. N. Walker, 80.
Dickey, Dr. J. S.—Applied anatomy of the lungs and pleural membranes, 263.
Ear, Diseases of the—Drs. W. Milligan and W. Wingrave, 167.
Ehrlich and S. Hata, P.—The experimental chemotherapy of spirilloses, 77.
Ely, Dr. L. W.—Joint tuberculosis, 163.
Emergencies of general practice—Drs. P. Sargent and A. E. Russell, 269.
Fearis, Dr. W. H.—The treatment of tuberculosis by means of immune substances, 359.
Feeble-mindedness in children of school age—Dr. C. P. Lapage, 81.
Fernie, Dr. W. T.—Health to date, 269.
First-aid charts—Dr. E. C. B. Ibotson, 173.
Fordyce, Dr. H. D.—The care of infants and young children, 271.
Fortescue-Brickdale, E. W. H. Groves and Dr. J. M.—Text-book for nurses, 365.
Fractures by mobilisation and massage, Treatment of—Dr. J. B. Mennell, 78.
Griffiths, Dr. F. G.—Studies in pulmonary tuberculosis, 268.
Groves, E. W. H.—A synopsis of surgery, 172.
Groves and Dr. J. M. Fortescue-Brickdale, E. W. H.—Text-book for nurses, 365.
Gynæcology, Principles of—Dr. W. B. Bell, 363.
Harris, Dr. A.—The etiology, diagnosis and prophylaxis of pulmonary phthisis, 267.
Hata, P. Ehrlich and S.—The experimental chemotherapy of spirilloses, 77.
Health to date—Dr. W. T. Fernie, 269.
Heredity, Some points in—Dr. R. C. Lucas, 270.
Hutchison and H. Rainy, Drs. R.—Clinical methods, 270.
Hygiene and public health—Sir A. Whitelegge and Sir G. Newman, 80.

**Books, Reviews of** (*continued*)—
Ibotson, Dr. E. C. B.—First-aid charts, 173.
I. K. therapy, Treatment of tuberculosis by—Dr. W. H. Fearis, 359.
Infants and young children, The care of—Dr. H. D. Fordyce, 271.
Inoculation, An introduction to therapeutic—Dr. D. W. C. Jones, 82.
Internal secretion and the ductless glands—Dr. S. Vincent, 361.
Joint tuberculosis—Dr. L. W. Ely, 163.
Jones, Dr. D. W. C.—An introduction to therapeutic inoculation, 82.
Kenwood and W. G. Savage, Drs. H. R.—Public health laboratory work, 79.
Lapage, Dr. C. P.—Feeble-mindedness in children of school age, 81.
Lockwood, C. B.—Clinical surgery, 172.
Love, Dr. J. K.—The deaf child, 266.
Low, Dr. R. C.—Carbonic-acid snow as a therapeutic agent, 82.
Lucas, Dr. R. C.—The Bradshaw lecture, 270.
Lungs, Applied anatomy of the—Dr. J. S. Dickey, 263.
Lyle, Dr. H. W.—Manual of physiology, 83.
Macdonald, Dr. D. M.—Ambulance examination questions, 83.
Macewen, W.—The growth of bone, 161.
McHattie, Dr. T. J. T.—The educational treatment of stammering children, 270.
Marie, Dr. A. [Ed.]—Traité international de psychologie pathologique, 362.
Marshall, Dr. B.—A manual of midwifery, 160.
Martindale and Dr. W. W. Westcott, W. H.—Salvarsan, 77.
Massage, Treatment of fractures by—Dr. J. B. Mennell, 78.
Medicine, A system of—Sir T. C. Allbutt and Dr. H. D. Rolleston, 74.
Melville-Davison, Dr. W.—Ships' hygiene, 162.
Mennell, Dr. J. B.—The treatment of fractures by mobilisation and massage, 78.
Merck's annual report, 83.
Midwifery, A manual of—Dr. B. Marshall, 160.
Miles, A. Thomson and A.—Manual of surgery, 171.
Milligan and W. Wingrave, Drs. W.—Diseases of the ear, 167.
Minchin, Dr. W. C.—Treatment of tuberculosis and lupus with allyl sulphide, 271.
Morland, Drs. C. Riviere and E.—Tuberculin treatment, 168.
Morris, Sir M.—Diseases of the skin, 273.
Mortimer, Dr. J. D.—Anæsthesia and analgesia, 160.
Mülzer, Dr. P.—The therapy of syphilis, 271.
Newman, Sir A. Whitelegge and Sir A.—Hygiene and public health, 80.
Nurses, Text-book for—E. W. H. Groves and Dr. J. M. Fortescue-Brickdale, 365.
Parsons and Dr. W. Wright, F. G.—Practical anatomy, 262.
Phthisis, Etiology, diagnosis and prophylaxis of pulmonary—Dr. A. Harris, 267.
Physics, Manual of—H. Candy, 83.
Physiology, Manual of—Dr. H. W. Lyle, 83.
Pickerill, Dr. H. P.—The prevention of dental caries and oral sepsis, 273.
Psychologie pathologique, Traité international de—Dr. A. Marie [Ed.], 362.

**Books, Reviews of** (*continued*)—
Public health laboratory work—Drs. H. R. Kenwood and W. G. Savage, 79.
Rainy, Drs. R. Hutchison and H.—Clinical methods, 270.
Rawling, Dr. L. B.—Landmarks and surface markings of the human body, 80.
Riviere and E. Morland, Drs. C.—Tuberculin treatment, 168.
Rolleston, Sir T. C. Allbutt and Dr. H. D.—A system of medicine, 74.
Russell, Drs. P. Sargent and A. E.—Emergencies of general practice, 269.
Salvarsan—W. H. Martindale and Dr. W. W. Westcott, 77.
Salvarsan, The treatment of syphilis with—Dr. W. Wechselmann, 77.
Sargent and A. E. Russell, Drs. P.—Emergencies of general practice, 269.
Savage, Drs. H. R. Kenwood and W. G.—Public health laboratory work, 79.
Schönwerth, Dr. A.—Chirurgisches vade-mecum, 172.
Ships' hygiene—Dr. W. Melville-Davison, 162.
Skin, Diseases of the—Sir M. Morris, 273.
Smith, J. S. K.—Lateral curvature of the spine, 269.
Spinal cord, Diseases of the—Dr. R. T. Williamson, 80.
Spine, Lateral curvature of the—J. S. K. Smith, 269.
Spirilloses, The experimental chemotherapy of—P. Ehrlich and S. Hata, 77.
Stammering children, The educational treatment of—Dr. T. J. T. McHattie, 270.
Surface markings of the human body—Dr. L. B. Rawling, 80.
Surgery, Clinical—C. B. Lockwood, 172.
Surgery, Manual of—A. Thomson and A. Miles, 171.
Surgery, Minor—L. A. Bidwell, 161.
Surgery, Synopsis of—E. W. H. Groves, 172.
Surgery, System of—Dr. C. C. Choyce, 74.
Surgical applied anatomy—Sir F. Treves, 173.
Surgical treatment, A manual of—Sir W. W. Cheyne and F. F. Burghard, 162.
Syphilis, The therapy of—Dr. P. Mülzer, 271.
Syphilis with salvarsan, The treatment of—Dr. W. Wechselmann, 77.
Thomson and A. Miles, A.—Manual of surgery, 171.
Thyroid therapy, Theory and practice of—H. E. Waller, 81.
Treves, Sir F.—Surgical applied anatomy, 173.
Tubby, A. H.—Deformities, including diseases of the bones and joints, 264.
Tuberculin treatment—Drs. C. Riviere and E. Morland, 168.
Tuberculosis and lupus with allyl sulphide, Treatment of—Dr. W. C. Minchin, 271.
Tuberculosis, Joint—Dr. L. W. Ely, 163.
Tuberculosis, Studies in pulmonary—Dr. F. G. Griffiths, 268.
Tuberculosis, Treatment of, by immune substances—Dr. W. H. Fearis, 359.
Tuberculous diseases of bones and joints—Sir W. W. Cheyne, 75.
Vincent, Dr. S.—Internal secretion and the ductless glands, 361.
Walker, Dr. N.—An introduction to dermatology, 80.
Waller, H. E.—Theory and practice of thyroid therapy, 81.

**Books, Reviews of** (*continued*)—
Wechselmann, Dr. W.—The treatment of syphilis with salvarsan, 77.
Westcott, W. H. Martindale and Dr. W. W.—Salvarsan, 77.
"Where the world ends," 81.
Whitelegge and Sir G. Newman, Sir A.—Hygiene and public health, 80.
Williamson, Dr. R. T.—Diseases of the spinal cord, 80.
Wingrave, Drs. W. Milligan and W.—Diseases of the ear, 167.
Wright, F. G. Parsons and Dr. W.—Practical anatomy, 262.
Breast, The differential diagnosis of swelling in the—C. A. Morton, 121.
Bristol Medico-Chirurgical Society, 89, 187, 282, 376.
Bristol medical library—C. K. Rudge, 344.
Bullen, F. St. J.—Some remarks on insanity, 205.

Carrick, Dr. A., and Bristol Medical Library Society, 174.
Carwardine, T.—Report on surgery, 257.
Chitty, H.—The artificial production of pneumothorax in phthisis, 141.
Clarke, Dr. J. M.—A case of chronic interstitial pancreatitis of uncertain origin, 25; Intra-cranial complications of ear disease, 97.
Constipation, Dietary treatment of, 353.
Coombs, Dr. C.—Report on medicine, 64.
Cotton, Dr. W.—The right and the wrong side of the X-ray picture: has a mistake been made? 227.
Cranial complications of ear disease, Intra- —Drs. J. M. Clarke and J. L. Firth, 97.

Davies, Dr. D. S.—An enemy of the people—tuberculosis and natural selection, 135.
Davis, Dr. O. C. M.—The relationship between toxicity and chemical reactivity in certain benzene derivatives, 237.
Diagnosis in acute intestinal obstruction, Some cases illustrating the difficulty and importance of early—E. W. Hey Groves, 37.
Diphtheria, Pseudo-, due to diplococcus crassus, 90.
Diseases bearing names of saints—Dr. R. Fletcher, 295.
Dreams and their significance, Notes on—Dr. G. H. Savage, 156.
Duodenum, Direct medication of, 353.

Eadon, W. F. B.—Obituary notice of, 285.
Ear disease, Intra-cranial complications of—Drs. J. M. Clarke and J. L. Firth, 97.
Edgeworth, Drs. E. L. Lees and F. H.—Notes on an unusual case of Hodgkin's disease, 150.
Editorial notes, 84, 174, 274, 367.
Education, The problem of medical—Dr. W. C. Swayne, 316.
Evans, U. W., Obituary notice of, 287.

Feeble-minded, Association for the permanent care of the, 177.
Female generative organs, Pain in diseases of, 283.
Fibromyomata of uterus causing obscure symptoms, 73.
Firth, Dr. J. L.—Intra-cranial complications of ear disease, 97.
Fletcher, Dr. R.—On some diseases bearing names of saints, 295.
Fletcher, Dr. R., Obituary notice of—Sir W. Osler, 289; editorial notes, 191, 367.
Fortescue-Brickdale, Dr. J. M.—Report on medicine, 350.
Fractures treated by intra-medullary pegs, 188.
Fuller, J., Obituary notice of, 383.

Gall-bladder cases presenting features of interest—Dr. E. Stack, 49.
Gastric analysis, 251.
Gastric super-acidity, Occurrence and treatment of, 381.
Glycyl tryptophane test, 254.
Groves, E. W. H.—Some cases illustrating the difficulty and importance of early diagnosis in acute intestinal obstruction, 37; Report on surgery, 68.

Heart-block occurring in acute rheumatism, 92.
Heroin habit, Case of—Dr. J. O. Symes, 153.
Hodgkin's disease, An unusual case of—Drs. E. L. Lees and F. H. Edgeworth, 150.

Immunity, Tuberculin and—Dr. J. W. Taylor, 338.

Insanity, Some remarks on—F. St. J. Bullen, 205.
Intestinal obstruction, Cases illustrating the difficulty and importance of early diagnosis in acute—E. W. H. Groves, 37.
Irido-cyclitis, 261.

Knee-joint, End-results of 41 operations for internal derangements of the—Dr. A. R. Short, 52.

Laryngoscopy, bronchoscopy, and œsophagoscopy, Direct—Dr. P. Watson-Williams, 11.
Lees and F. H. Edgeworth, Drs. E. L.—Notes on an unusual case of Hodgkin's disease, 150.
Leprosy in South Africa, 192.
Leptothrix blood infection, Fever due to, 89.
Library, Bristol medical—C. K. Rudge, 344.
Library of the Bristol Medico-Chirurgical Society, 87, 184, 280, 344, 373; Annual report, 376.
Lister: an appreciation—Dr. R. Roxburgh, 1.
Local medical notes, 94, 191, 287, 383.

Mariette, Dr. E.—Personal experience of pneumothorax in phthisis, 189.
Maurice, O. C., Death of, 181.
Medicine, Reports on — Dr. C. Coombs, 64; Dr. J. M. Fortescue-Brickdale, 350; Dr. J. A. Nixon, 251.
Miner's nystagmus, 259.
Morton, C. A.—The differential diagnosis of swelling in the breast, 121.

Nixon, Dr. J. A.—Report on medicine, 251.
Nose and pharynx, Tuberculosis of the—Dr. A. J. Wright, 45.

Obituary, 93, 285, 289, 382.
Obstetrics, Report on — D. C. Rayner, 72.
Ogilvy, Dr. A.—Report on ophthalmology, 259.
Ophthalmology, Report on—Dr. A. Ogilvy, 259.
Osler, Sir W.—Robert Fletcher, 289.

Pain, Causes of chronic recurrent abdominal, 282.
Palate, innervation of the, 188.
Pancreatitis, Case of chronic interstitial—Dr. J. M. Clarke, 25.
Parker, Dr. G.—The barber surgeons, 327.
Parsons, J. F., Obituary notice of, 93.
Pericarditis, 64.
Phthisis, Artificial production of pneumothorax in — H. Chitty, 141.
Phthisis, Personal experience of pneumothorax in — Dr. E. Mariette, 189.
Pituitrin, Use in obstetrics, 72.
Pneumothorax, Cases of, 90.
Pneumothorax, Artificial production of in phthisis—H. Chitty, 141; Personal experience of — Dr. E. Mariette, 189.
Precocious development, Case of, 188.
Pregnancy, Operations on the uterus and appendages during—Dr. W. C. Swayne, 31.
Prichard, Dr. J. C., and Bristol Medical Library Society, 174.
Pyloric stenosis, Congenital, 378.
Pyocyaneus septicæmia, Case of, 379.

Raynaud's disease—Dr. D. A. Alexander, 247.
Rayner, D. C.—Report on obstetrics, 72.
Rheumatic anthritis in a child, 380.
Roxburgh, Dr. R.—Lister: an appreciation, 1; Tuberculosis, 193.
Rudge, C. K.—The Bristol medical library 344.

Saints, On some diseases bearing names of—Dr. R. Fletcher, 295.
Savage, Sir G. H.—Notes on some dreams and their significance, 156.
Scrofulosis, 261.
Short, Dr. A. R.—The end-results of 41 operations for internal derangements of the knee-joint, 52.

**Sick, Preparations for the:**
Agarase, 86.
Asensitine, 279.
Atophan-Schering, 371.
Balmosa, 278.
Cascaromat, 86.
Codeonal, 372.

**Sick, Preparations for the :** (*con.*)
Collosal argentum, 85, 181.
,, hydrargyrum, 85, 181.
Cystopurin, 371.
Dental hypodermic syringe, 183.
Digipuratum, 182.
Elixir pro pertussi, 279.
Eucerinum, 182.
Gelatine capsules, Flexible, 279.
Glaxo, 276.
Glycerole lecithin, 278.
Guttaplasts, 182.
Hydrastinin-hydrochl, 279.
Hyposal ampoule-syringe, 277.
Iodex c methyl salicylat, 183.
Kaloof compress, 372.
Kerol, 276.
Laibose, 276.
Leucoplasts, 182.
Levico water, 277.
Malt extract with iron iodide, 278.
Malt-glidine, 371.
Milk-chocolate peptonised, 372.
Neutrigen tonic food, 372.
Nivea soap, 182.
Ovogal, 86.
Palatinoid carophylli pulv., 278.
Paroleine spray compound, 370.
Peristaltine ciba, 86.
Pituitary extract, 279.
Pond's plasters, 183.
Sal antisepticus, 86.
Sapo antisepticus etherius, 279.
Tabloid epinine compound, 370.
,, hypodermic ergamine, 369.
,, ophthalmic physostigmine salicylate, 370.
,, ophthalmic pilocarpine nitrate, 370.
Thaolayine, 86.
Tromner elixir of cod liver oil, 181.
Unguentum iothion, 279.
Ureabromin, 182.
Vaginal douche tablets, 279.
Vaporole ammonium chloride inhaler attachment, 84.
Zematone, 183.
Siddall, Dr. J. B., 84.
Sinus thrombosis, Three cases of lateral, 91.
Smith, Dr. R. S., Resignation as editor, 369, 377.
Society, Bristol Medico-Chirurgical, 89, 187, 282, 376.
Stack, Dr. E.—Some gall-bladder cases presenting features of interest, 49.
Stoke-Adams syndrome, 89.
Stomach, Ulcers of, surgery of, 68.
Stomach, Ulcers of, Symptomatology of, 69, 255, 351.
Stomach, Ulcers of, Treatment of acute perforating, 257.
Surgery, Reports on — T. Carwardine, 257 ; E. W. H. Groves, 68 ; C. F. Walters, 354.
Suture of arteries, 354.
Swayne, Dr. W. C.—Operations on the uterus and appendages during pregnancy, 31 ; The problem of medical education, 316.
Symes, Dr. J. O.—A case of the heroin habit, 153.
Syphilitic eye affections, 261.

Tayler, G. C., Obituary notice of, 382.
Taylor, Dr. J. W.—Tuberculin and immunity, 338.
Tuberculin and immunity—Dr. J. W. Taylor, 338.
Tuberculin and tuberculosis, 180.
Tuberculosis—Dr. R. Roxburgh, 193.
Tuberculosis and natural selection—Dr. D. S. Davies, 135.
Tuberculosis of the nose and pharynx—Dr. A. J. Wright, 45.
Tuberculosis officer, The whole-time, 274.

Ulcers of the stomach and duodenum, Acute perforating, 257.
Ulcers of the stomach and duodenum, Symptomatology of, 69, 255, 351.
Uterine operations during pregnancy—Dr. W. C. Swayne, 31.
Uterus, Fibromyomata of, Obscure symptoms caused by, 73.

Vascular system, Surgery of the, 354.

Walters, C. F.—Report on surgery, 354.
Watson-Williams, Dr. P.—Direct laryngoscopy, bronchoscopy, and œsophagoscopy, 11.
Wright, Dr. A. J.—Some observations on tuberculosis of the nose and pharynx, 45.

X-ray picture, The right and wrong side of the—Dr. W. Cotton, 227.
X-rays in gastric diagnosis, 70, 253, 350.

LE My '13

### *PUBLICATIONS RECEIVED.*

From Adlard and Son, London:
*Deuxième Congrès de l'Association Internationale d'Urologie.* 1911. *Procès-Verbaux.* 1912. Price, 10s. net.

From Edward Arnold, London:
*Internal Secretions and the Ductless Glands.* By Swale Vincent, M.D., D.Sc. 1912. Price, 12s. 6d. net.
*Forensic Medicine and Toxicology.* By C. O. Hawthorne, M.D. Third Edition. 1912. Price, 6s. net.

From Baillière, Tindall and Cox, London:
*The Clinical Pathology of Syphilis and Parasyphilis.* By Hugh Wansy Bayly, M.A., M.R.C.S. 1912. Price, 5s. net.
*The Nutrition of the Infant.* By Ralph Vincent, M.D. Fourth Edition. 1913 [*sic*]. Price, 10s. 6d. net.

From John Bale, Sons and Danielsson, Ltd., London:
*Studies in Clinical Medicine.* By C. O. Hawthorne, M.D. 1912. Price, 6s. net.

From Cassell and Company, Ltd., London:
*A System of Surgery.* Edited by C. C. Choyce, B.Sc., M.D., F.R.C.S. Pathological Editor: J. Martin Beattie, M.A., M.D., C.M. Volume II. 1912. Price 21s. net.

From J. & A. Churchill, London:
*The Treatment of Ringworm.* By J. H. Sequeira, M.D. 1912. Price, 1s. net.

From Cornish Bros., Birmingham:
*Insomnia: its Causes and Treatment.* By Sir James Sawyer. Second Edition. 1912.

From Henry Frowde and Hodder and Stoughton, London:
*The Surgery of the Skull and Brain.* By L. Bathe Rawling, F.R.C.S. 1912. Price, 25s. net.
*Bacteria.* By Dr. Max Schottelius. Second Edition. Translated by Staff-Surgeon Herbert Geoghegan, R.N. 1912. Price, 8s. 6d. net.
*Manual of Medicine.* By A. S. Woodwark, M.D. 1912. Price, 10s. 6d. net.

From Charles Griffin and Company, London:
*Clinical Medicine.* By Judson S. Bury, M.D. Third Edition. Edited by Judson S. Bury and Albert Ramsbottom, M.D. 1912. Price, 17s. 6d. net.

From P. S. King and Son, London:
*The Causes leading to Educational Deafness in Children.* By Macleod Yearsley, F.R.C.S. 1912. [Reprint.] Price, 1s.
*Hygiene for Health Visitors, School Nurses and Social Workers.* By C. W. Hutt, M.A., B.C. D.P.H. 1912. Price, 7s. 6d. net.
*Third Report of Deptford School Clinic or Health Centre, for* 1911–12. 1912.
*Medical Benefit in Germany and Denmark.* By I. G. Gibbon, B.A., D.Sc. 1912. Price, 6s. net.

From J. F. Lehmann, Munich:
*Nierentuberkulose.* Von Dr. Felix Schlagintweit. 1912. Price, M. 4.

From H. K. Lewis, London:
*Elements of Practical Medicine.* By Alfred H. Carter, M.D., M.Sc. Tenth Edition. 1912. Price, 9s. net.
*The Extra Pharmacopoeia of Martindale and Westcott.* Revised by W. Harrison Martindale and W. Wynn Westcott. Fifteenth Edition. 2 vols. 1912. 21s. net.
*Clinical Bacteriology and Hæmatology for Practitioners.* By W. D'Este Emery, M.D., B.Sc. Fourth Edition. 1912. Price 7s. 6d. net.
*Materia Medica and Pharmacy.* By Reginald R. Bennett, B.Sc., F.I.C. Second Edition. 1912. Price, 4s. 6d. net.
*Mind and its Disorders.* By W. H. B. Stoddart, M.D., F.R.C.P. Second Edition. 1912. Price, 12s. 6d. net.

From E. & S. Livingstone, Edinburgh:
*Public Health Law.* By William Robertson, M.D., D.P.H., and Archibald McKendrick, D.P.H. 1912. Price, 5s. net.
*Extraction of Teeth.* By J. H. Gibbs, F.R.C.S., L.D.S.Edin. 1912. Price, 7s. 6d. net.

From Longmans, Green and Co., London:
*A Manual of Surgical Treatment.* By Sir W. Watson Cheyne, Bart., C.B., and F. F. Burghard, M.S., F.R.C.S. New edition, entirely revised and rewritten with the assistance of T. P. Legg, M.S., F.R.C.S., and Arthur Edmunds, M.S., F.R.C.S. Vol. III. 1912. Price, 21s. net.

From James Maclehose and Sons, Glasgow:
*Life of Sir William Tennant Gairdner, K.C.B., M.D., LL.D., F.R.S.* By George Alexander Gibson, M.D., Sc.D., LL.D. 1912. Price, 10s. 6d. net.
*A Manual of Immunity.* By Elizabeth T. Fraser, M.D. 1912. Price, 5s. net.

From Macmillan and Co. Ltd., London:
*A Text-Book of Pathology.* By J. George Adami, M.A., M.D., and John McCrae, M.D. 1912. Price, 25s. net.
*Anæsthetics and their Administration.* By Sir Frederic W. Hewitt, M.V.O., M.A., M.D. Fourth edition, prepared with the assistance of Henry Robinson, M.A., M.D., B.C. 1912. Price, 15s. net.
*Diseases of the Liver, Gall-Bladder and Bile-Ducts.* By Humphry Davy Rolleston, M.A., M.D. [Second Edition.] 1912. Price, 25s. net.

From John Murray, London:
*The Treatment of Tuberculosis by means of Immune Substances (I.K.) Therapy.* By Walter H. Fearis, M.D. 1912. Price, 6s. net.

From Stanley Paul and Co., London:
*The First Signs of Insanity: their Prevention and Treatment.* By Bernard Hollander, M.D. [1912.] Price, 10s. 6d. net.

From Puccio y Muhlrad, Buenos Ayres:
*Anuario médico Sud-Americano.* 1912.

From Snow and Farnham Company, Providence (R.I.):
*Medical Inspection of Schools.* By Allen G. Rice, M.D. 1912.

From Stephen Swift and Co. Ltd., London:
*The Doctor and his Work.* By Charles J. Whitby, M.D. 1912. Price, 3s. 6d. net.

From John Wright & Sons Ltd., Bristol:
*Manual for Women's Voluntary Aid Detachments.* By P. C. Gabbett, M.R.C.S., Lt. Col. I.M.S. [N.D.] Price, 1s. net.
*An Historical Outline of Ambulance from the Earliest Times.* By Charles H. Miles, L.R.C.P. [1912.] Price, 3d.
*Small-pox and its Diffusion.* By Alexander Collie, M.D. 1912. Price, 2s. net.
*Statistics of Puerperal Fever and Allied Infectious Diseases.* By George Geddes, M.D., C.M. 1912. Price, 6s. net.
*Diseases of the Throat, Nose, and Ear.* By W. G. Porter, M.B., B.Sc. 1912. Price, 7s. 6d. net.
*A Manual of Infectious Diseases occurring in Schools.* By H. G. Armstrong, M.R.C.S., and J. M. Fortescue-Brickdale, M.A., M.D. 1912.

